# The Effectiveness of a Wireless Modular Bed Absence Sensor Device for Fall Prevention among Older Inpatients

**DOI:** 10.3389/fpubh.2016.00292

**Published:** 2017-01-09

**Authors:** Kogilavani Subermaniam, Ridgwan Welfred, Pathmawathi Subramanian, Karuthan Chinna, Fatimah Ibrahim, Mas S. Mohktar, Maw Pin Tan

**Affiliations:** ^1^Anatomy and Physiology Unit, Allied Health Science College, Ministry of Health Malaysia, Sungai Buloh, Malaysia; ^2^Department of Biomedical Engineering, Faculty of Engineering, University of Malaya, Kuala Lumpur, Malaysia; ^3^Centre for Innovation in Medical Engineering (CIME), Faculty of Engineering, University of Malaya, Kuala Lumpur, Malaysia; ^4^Department of Nursing Science of Faculty of Medicine, University of Malaya, Kuala Lumpur, Malaysia; ^5^Ageing and Age-Associated Disorders Research Group, Faculty of Medicine, University of Malaya, Kuala Lumpur, Malaysia; ^6^Social and Preventive Medicine of Faculty of Medicine, University of Malaya, Kuala Lumpur, Malaysia; ^7^Division of Geriatric Medicine, Faculty of Medicine, University of Malaya, Kuala Lumpur, Malaysia

**Keywords:** accidental fall, aged, clinical alarm, nurses, preventive measure

## Abstract

**Background:**

Falls and fall-related injuries are increasingly serious issues among elderly inpatients due to population aging. The bed-exit alarm has only previously been evaluated in a handful of studies with mixed results. Therefore, we evaluated the effectiveness of a modular bed absence sensor device (M-BAS) in detecting bed exits among older inpatients in a middle income nation in East Asia.

**Methods:**

Patients aged ≥65 years on an acute geriatric ward who were able to mobilize with or without walking aids and physical assistance were recruited to the study. The total number of alarms and the numbers of true and false alarms were recorded by ward nurses. The M-BAS device is placed across the mattress of all consenting participants. Nurses’ workload was assessed using the National Aeronautics and Space Administration-Task Load Index (NASA-TLX) score, while nurses’ perceptions were surveyed.

**Results:**

The sensitivity of the M-BAS was 100% with a positive predictive value of 68% and a nuisance alarm rate of 31%. There was a significant reduction in total NASA-TLX workload score (mean difference = 14.34 ± 13.96 SD, *p* < 0.001) at the end of the intervention period. 83% of the nurses found the device useful for falls prevention, 97% found it user friendly, and 87% would use it in future.

**Conclusion:**

The M-BAS was able to accurately detect bed absence episodes among geriatric inpatients and alert nurses accordingly. The use of the device significantly reduced the total workload score, while the acceptability of the device was high among our nurses. A larger, cluster randomized study to measure actual falls outcome associated with the use of the device is now indicated.

## Introduction

Patient safety is paramount in order to achieve quality health care. Adverse events such as falls, alongside mortality and morbidity, are considered as negative outcomes associated with poor quality of care (1). Falls have been reported to be among the most common type of inpatient accidents (2, 3), which compromises patient safety in health-care institutions. The increasing incidence of falls alongside the rapidly growing older population worldwide incurs direct and indirect costs, which lead to serious social and economic consequences. In addition, falls are also associated with serious psychological consequences. Fifty-four percent of individuals aged 70 years and above express fear of falling, which results in reduction in physical and social activities, which then leads to loss of independence and social engagement (4).

Falls occur as a result of the complex interplay between predisposing or precipitating factors, which could be intrinsic or extrinsic in nature (5, 6). The environment, demographic factors, clinical characteristics, and medications therefore often all contribute synergistically to falls in the older person. Hospitalization is an important risk factor for falls due to the change in environment, the drastic disruptions in life habit that occur with being in a regimented environment with constant unexpected interruptions in the daily routine, and underlying patient factors such as acute illness and delirium or cognitive impairment (7).

While numerous effective fall prevention strategies have been established for older individuals in the community, the evidence behind falls prevention among older inpatients remains inadequate; with few effective intervention strategies currently available (6). Bed-exit alarms have been advocated among older inpatients perceived to have increased risk for falls. The results of the few studies that have evaluated the effectiveness of these alarms have been conflicting with the most recent large, randomized-controlled study showing no reduction in falls with the use of a bed-sensor alarm (8). However, the effectiveness of the bed-exit monitor in preventing falls rely on numerous factors, including the design of the alarm sensors, the likelihood of health-care workers responding to the alarms, as well as the selection of patients.

Previously employed strategies of identifying hospitalized patients in whom fall prevention strategies should be targeted are of unclear benefit. In particular, falls risk assessment among hospital inpatients is fraught with controversy, as it has been demonstrated that most falls occur in individuals categorized as low risk using the tool, leading to the recently published National Institute for Health and Clinical Excellence guidelines now no longer advocating the use of falls risk assessment tools (9). Instead, they recommended that all older individuals should be considered at high risk of falls. However, the proposed universal approach to fall prevention will lead to additional staff burden in an already overstretched health-care systems.

In light of the recent revelations in falls prevention in older inpatients, we conducted a study to examine the effectiveness of a wireless modular bed absence sensor device (M-BAS) as a fall prevention strategy among older inpatients. The aim of our study was to determine the effectiveness of the device in alerting nurses to bed-exit episodes, to determine the effect of the introduction of the device on ward nurses’ workload, as well as nurses’ perception on the usefulness of the device in preventing falls. Studies of this nature are also rarely conducted in lower to middle income settings like ours. Hence, our study will also be providing new information on the use of assistive technology in non-high income countries.

## Materials and Methods

### Study Design

This was a two-part study, employing an uncontrolled design evaluating the effectiveness of the M-BAS in identifying bed-exit events and the nurses’ perception on the usefulness of the device, as well as a quasi-experimental design comparing the workload of nurses before and after the introduction of the M-BAS.

#### Ethical Considerations

Ethical approval for conducting the study was granted by the University Malaya Medical Centre (UMMC) Medical Ethics Committee on November 28, 2013 (MEC ID No.: 201311-0479) and complied fully in accordance to the Declaration of Human Rights, Helsinki, 1975. Data were collected upon approval. Three aspects of ethical consideration that included informed consent, anonymity and confidentiality, and permission to use the tool were discussed.

#### Informed Consent

All respondents (patients and nurses) in the study participated through written informed consent. A letter explaining the purpose of the study, contact number to call, and how their anonymity together with their confidentiality were assured and protected were given to the respondents. At the same time, the respondents were informed of their rights to reject participation and withdraw at any time during the study period.

#### Anonymity and Confidentiality

Respondents’ information was identified with unique codes to maintain anonymity. The unique identifier key was kept locked in a locked drawer in a secure location, and all completed questionnaires were stored in a secured location. All documents will be kept for at least 7 years.

### Participants

#### Patients

Consecutive patients admitted to the acute geriatric ward at a large teaching hospital during a 2-month period from January to March 2014 were considered for the study. The total admission in the ward from January 1 to March 31, 2014, was 209. However, according to the study period for the patients which started from January 28 to 31, 2014, it was only 156 patients admitted to the ward. Throughout the study period, the researcher surveyed the number of patients who were eligible for the study and it was only 47 patients. This was based on the inclusion criterion; age 65 years and above and able to mobilize with or without a walking aid. Patients were excluded if they were bedfast. Written informed consent was obtained from each participant or their next of kin prior to enrollment into the study.

#### Nurses

All the staff nurses (30) who worked in the same concerned geriatric ward of UMMC during the study period from January 13 to April 16, 2014, were the target population and were recruited in the study.

### The Modular Bed Absence Alarm Device

The modular bed absence or bed-exit device (patent pending) consisted of a thin sensing pad made of flexible material placed beneath the patients back underneath the bed coverings. The sensor differs from previously marketed devices in its modular design. It consisted of three panels to be placed across the width of the bed. Pressure on the central panel will silence the alarm, while pressure on the two side panels will trigger an intermittent alarm. A loud, high-pitched alarm will be triggered if no pressure is applied across all three panels to raise immediate attention that the patient has left the bed. The battery powered sensor is connected wirelessly to a battery operated palm-sized receiver, which can be carried around by the nurse while she performs her duty. The original prototype of the sensor alarm had been field tested and refined prior to the commencement of the study.

### Intervention

The sensor pad was positioned on the beds of all participants from the time of recruitment until they were discharged from the hospital. Nurses caring for the participants were asked to record all alarm episodes and whether they were true alarms or false alarms in a simple form attached to the patient’s observation chart. All ward nurses received training on how to use the device and how to log the alarm activity. The researcher attended the ward on a daily basis to ensure that the device was being applied appropriately and the logs were completed accurately.

### Nurses’ Workload

The nurses’ workload was assessed before and after the intervention using the National Aeronautics and Space Administration-Task Load Index (NASA-TLX) questionnaire (10). The NASA-TLX questionnaire consisted of six subscales, namely mental demand, physical demand, temporal demand, performance, effort, and frustration. The lowest score for each subscale was 5 and the highest 100. A separate weighted scoring for the source of workload (weight of workload) was also calculated. The minimal score for each source of workload was 0, and maximal score for each source was 5. The maximal total score for source of workload was 15. The total workload score was then calculated based on the raw subscale and source of workload scores.

### Acceptability

A 12-item survey was also administered at the end of the study to determine the acceptability of the device by ward nurses. The survey was pretested on geriatricians, clinical specialists, and nurses on a postgraduate course prior to administration. Nurses’ perception on the usefulness of the device, effects of workload, ease of use, and future usage were assessed with the questionnaire survey.

### Statistical Analysis

Participants’ demographic data were expressed as frequencies and percentages for categorical variables. For the numerical variables, normally distributed variables were presented as mean ± SD, while non-normally distributed variables were presented as median ± interquartile range (IQR). The total workload scores measured before and after the intervention were assessed using the paired *t*-test, while individual subscale scores were compared using the Wilcoxon signed-rank test. The Mann–Whitney *U* test was used to compare the number of true and false alarms per patient per day according to patient characteristics. A *p*-value of <0.05 was considered statistically significant. The five point Likert scales for the survey responses were dichotomized into agreed or strongly agreed (4 and 5) and not sure or disagree (1 to 3). All data were analyzed using Statistical Package for Social Science (SPSS) version 20.0 software (SPSS, Chicago, IL, USA).

## Results

One hundred and fifty-six patients were admitted to the acute geriatric ward during the study period. 47 of the 156 (30.1%) fulfilled the recruitment criteria. Thirty-one patients (*n* = 31) out of 47 patients who met the inclusion criteria (66.0%) agreed to participate in the study. The baseline characteristics of participants are summarized in Table 1. Eighteen patients (58.1%) had a history of falls over the past 12 months; 12 (38.7%) were diagnosed with neurological disorders namely depression, stroke, basal ganglia bleed, hemiparesis, syncopal attack, subdural hemorrhage, and seizures; the remainder had the diagnoses of respiratory, cardiovascular, genitourinary, fluid, and electrolyte or metabolic disorders.

**Table 1:** **Demographic characteristic of participants**.

Characteristic of participants	Mean/frequency (*n* = 31)	SD/%
Age (years), mean (SD)	83	7
Weight (kg), mean (SD)	57	7
Female gender, *n* (%)	19	61
Use of mobility device	11	36
Dementia	10	32
Delirium	3	10
History of fall	18	60

### True and False Alarm

The 31 participants used the M-BAS device for a total of 328 days. A total number 119 alarms were recorded. Eighty-one of the 119 (68%) alarms were true alarms, and 38 (32%) were false alarms. Out of the 81 true alarms, 79 (98%) were genuine bed-exit attempts, while two (2%) true alarms occurred while the nurses were performing manual transfers without first switching off the alarm. The sensitivity of the bed alarm device was therefore determined as 100% with a positive predictive value of 68%. The false positive rate for the alarm device was 31%. It was not possible to calculate the specificity of our device.

### Participant Characteristics versus Alarm Characteristics

The minimum number of days the device was applied to a patient was 2 and maximum was 44. The minimum number of alarm activity per patient was 0, while the maximal number of alarms per patient was 15. The number of true and false alarms were adjusted for differences in length of stay in individuals by calculating the number of alarms per patient per day. The mean ± SD for number of alarms per patient per day was 0.37 ± 0.34 alarms/day. The median ± IQR number of true alarms per patient per day was 0.14 ± 0.38 alarms/day while the median number of false positive alarms per patient per day 0.00 ± 0.20 alarms/day. Comparisons of the patients’ characteristic versus number of true and false positive alarms per patient per day did not reveal any significant differences in true and false alarm rates according to patient characteristics (Table 2).

**Table 2:** **Patient characteristics versus true and false alarms/patient/day**.

Patient characteristics	True alarms/patient/day			False alarms/patient/day		
	Median	IQR	*p*-value	Median	IQR	*p*-value
**Gender**						
Male	0.14	0.39	0.984	0.01	0.25	0.810
Female	0.20	0.38		0.00	0.17	
**Mobility device**						
Yes	0.09	0.71	0.983	0.00	0.17	0.841
No	0.17	0.32		0.01	0.25	
**Dementia**						
Yes	0.13	0.29	0.547	0.45	0.32	0.785
No	0.20	0.51		0.00	0.19	
**History of fall**						
Yes	0.16	0.43	0.639	0.01	0.18	1.000
No	0.14	0.36		0.00	0.23	

### Survey of Nurses’ Perception on Usefulness

All 30 ward nurses, aged, mean ± SD, 28 ± 5 years, 26 (87%) women, agreed to participate in our study. All nurses had a diploma in nursing with 11 (37%) having received an additional 6 months of post-basic gerontology training. The mean ± SD years of experience was 5 ± 4 years. Table 3 represents the summary table for the NASA-TLX workload subscale and total scores before and after the intervention program. There were statistically significant differences in the median score for mental and physical demand between pre and post-intervention periods (Table 3). Using the paired *t*-test, we also found a statistically significant difference in mean total workload scores between the pre and post-intervention periods (mean difference = 14.34 ± 13.96, *p* < 0.05) (Table 3).

**Table 3:** **Median or mean difference in workload subscale and total scores**.

Subscales	Wilcoxon signed ranks test paired differences		

	Median/mean difference	*Z*/*t*	*p*-Value
Mental	115	−2.693	**0.007**
Physical	95	−3.138	**0.002**
Temporal	15	−1.606	0.108
Performance	20	−0.498	0.619
Effort	15	−0.314	0.754
Frustration	37.5	−1.058	0.290
Total workload score	14.34	5.63	<0.001**

*Wilcoxon signed-rank unless otherwise indicated*.

*Text in bold represents statistical significance*.

**Paired t-test.

The nurses’ responses to the survey questionnaire are summarized in Table 4. Seventy-seven percent and 83% agreed that the device was useful for fall prevention and fall detection, respectively. Fifty-seven percent agreed that it reduced their workload. Ninety-seven percent agreed that the device used simple technology, while 90% agreed it was simple to use. Eighty-seven percent agreed they would use them for their patients and would encourage their friends to use them, while 83% agreed it was suitable for elderly patients.

**Table 4:** **Nurses’ perception on the usefulness of the wireless modular bed alarm device**.

Survey items		Agreed	Disagreed/not sure

		*n* (%)	*n* (%)
**Fall detection**			
Q1	Helped me to detect falls fast	25 (83.3)	5 (16.7)
Q2	Able to alert me accurately in regards to patients’ movement	15 (50)	15 (50)
Q3	Help me to manage my patient well in terms of fall prevention	23 (76.7)	7 (23.3)
**Workload**			
Q4	I do not have to be at the patients’ bed side always to monitor their movements	17 (56.7)	13 (43.3)
Q5	Provides me more time for other work	17 (56.7)	13 (43.3)
Q6	Helped reduce my work load	17 (56.7)	13 (43.3)
**Usage of the bed alarm**			
Q7	Able to alert me even I am away from the patients’ bed	24 (80.0)	6 (20.0)
Q8	Used simple technology and easy to operate/handle (user friendly)	29 (96.7)	1 (3.3)
Q9	It is easy to use	27 (90.0)	3 (10.0)
**Bed alarm use in future**			
Q10	Will use the bed alarm in future for my patient	26 (86.7)	4 (13.3)
Q11	Will encourage my colleague to use the bed alarm	26 (86.7)	4 (13.3)
Q12	It is suitable to be used for my elderly patients	25 (83.3)	5 (16.7)

## Discussion

Few falls prevention and falls detection devices have been evaluated using real patient data. Our study was also unique with its setting being within a middle income country. Our modular bed alarm system was able to alert ward nurses of bed exits with a sensitivity of 100% and an acceptable nuisance alarm rate of 32%. In addition, the total number of alarms per patient per day was only 0.3, which indicated that the alarm was only triggered around once every three days per patient. These figures are encouraging, as the primary concern of using a bed-exit alarm on a busy geriatric ward is alarm fatigue should the bed-exit alarm be triggered regularly. Increasing numbers of bedside monitors and other medical equipment are now being adopted for patient care, which leads to genuine concerns of alarm fatigue (11). However, the rate of alarm of our devices is relatively low, and the additional advantage of wireless technology allows the nurse to wear the receiver or place it at a suitable location, which therefore allows the nurse to differentiate the alarms from the bed-exit sensor from regular alarms from other medical devices.

A recent cluster randomized trial of an intervention to increase bed alarm use in hospital nursing units by Shorr et al. (8) reported that increased use of the bed-exit alarm had no statistically significant effect on the number or the rate of falls, injurious falls, or patients restrained in the intervention group compared with control units. The main aim of Shorr et al.’s (8) study was to determine whether an intervention to increase bed alarm use was effective in reducing falls rather than an evaluation of whether bed alarm use itself reduced falls, as control units also had access to bed alarms. The design of their bed alarm system was different, and their patient identification method relied on a falls risk assessment score, the problems with which were discussed earlier. Previous studies had also published their evaluation of bed-exit alarms in real patients, but their study focused (12) mainly on the evaluation of a dual sensor using infrared and pressure-sensitive alarms compared to pressure sensitivity alarms, and their study was conducted in only 14 nursing home residents. Our patient selection was that of universal sampling, where the M-BAS was applied to all consenting participants who were capable of mobilizing regardless of their falls risk score. Inpatient falls frequently occur when patients attempt to leave their beds, usually to go to the bathroom, unsupervised (13). Therefore, falls prevention strategies adopted by hospitals universally include demonstrating the use of the call bed and reminding patients of the need to call for assistance if they need to leave their beds. However, many patients still do not call for assistance despite these measures (14). This may occur due to patients’ reluctance to bother nurses, the presence of delirium or longer term cognitive decline or nurses not attending to their calls for assistance promptly, to name but a few plausible explanations. The use of a bed alarm system is therefore an additional safety measure to ensure that nurses are alerted to bed absences when the patient does not call for assistance.

The addition of further tasks to the nurses’ increasingly busy work schedule naturally raises concerns over nurses’ workload, which is becoming increasingly burdensome with the increasing age and comorbidities of their patient profiles and the introduction of increasingly complex medical and surgical interventions. Our study has however demonstrated objectively, an overall reduction in nurses’ workload with the introduction of the bed alarm. Individual subscale scores were also significant for reduction in mental and physical demand. While carer supervision is considered the most effective intervention for reducing falls in institutions, it is not possible for all older inpatients to be supervised constantly. Therefore, the M-BAS assists in the task of supervision and hence releases the mental and physical strain of attempting to provide supervision at all times.

The acceptability of bed-exit monitors in our setting was high. The nurses appeared convinced of the device in falls prevention and nearly 90% will continue using the device in future. The M-BAS device is an uncomplicated device that can be manufactured at minimal cost and therefore shows good potential in being utilized in resource limited settings such as the study site. Furthermore, its utility could also be extended to other clinical applications such as for the management of patients with delirium who are at risk of wandering. This latter application would help address human rights issues, as well as psychological and physical costs associated with the use physical restraints which are sadly rampant in our setting (15).

The universal use of our device in all patients who were able to mobilize embraces the notion that increased age alone is associated with increased risk of falls (9). As falls risk among patients is difficult to predict accurately (16), the universal approach does seem like the only effective way at the moment. The bed absence alarm is a simple piece of electronic device, which can be manufactured at a fraction of the cost of a hospital bed. Therefore, this approach appears to be feasible and well accepted by our ward nurses. A larger cluster randomized-controlled study should now be conducted to determine actual falls outcomes in hospitals and other institutions providing care for the elderly. While cluster randomization of the use of bed alarms compared to no bed alarms may no longer be possible in higher income countries, as bed alarms have already penetrated their markets for many years and are now widely available, the evaluation of the use of such technology in our setting remains possible with resource limitations being the only major barrier. To our knowledge, our hospital ward is the first unit in our country to employ the use of bed alarm systems.

The main limitations of our study are its short term design leading to the lack of actual falls outcomes and the possibility of reporting bias in obtaining information on alarm episodes. While falls are considered common in hospital patients, only one or two falls may occur per hospital bed in a year. Therefore, an adequately powered study to detect statistically significant differences in falls outcome will require far longer study periods and the enrollment of a large number of patients. Such a study may be financially too prohibitive in our setting but may no longer be possible in higher income countries as bed absence alarms are already widely used. There may be reporting bias for the detection of alarm events by our ward nurses. However, a researcher attended the ward daily, and the nurses were also provided with token rewards for completing the logs and the questionnaires, to ensure maximal participation and to minimize this bias.

## Conclusion

Our modular bed absence alarm system was effective in alerting nurses when patients were about to leave or had left their beds, with a sensitivity of 100% and an acceptable nuisance alarm rate of 32%. The total workloads as well as mental and physical subscale scores using the NASA-TLX score were significantly lower with the use of the M-BAS device in all patients who were able to mobilize. Our ward nurses felt that the M-BAS was effective in preventing falls, found the device easy to use, and were willing to use the device in the future, with over 50% also agreeing to it reducing their workload. A larger, cluster randomized-controlled study evaluating the universal use of the M-BAS on all ambulatory older patients in institutionalized settings should therefore now be considered.

## Author Contributions

Conceived and designed the experiments: KS, PS, and MT. Device invention and technical support: RW, FI, and MM. Performed the experiments: KS. Analyzed the data: KS, MT, and KC. Wrote the paper: KS, PS, and MT.

## Conflict of Interest Statement

MT has received honoraria from Novartis, Lundbeck, Boehringer Ingelheim, and Merck, Sharp & Dohme as a speaker to on dementia treatment, anticoagulation in atrial fibrillation, and adult vaccination. The other authors declare no conflict of interest.
